# Identification of macrophage migration inhibitory factor and human neutrophil peptides 1–3 as potential biomarkers for gastric cancer

**DOI:** 10.1038/sj.bjc.6605138

**Published:** 2009-06-23

**Authors:** Y Mohri, T Mohri, W Wei, Y-J Qi, A Martin, C Miki, M Kusunoki, D G Ward, P J Johnson

**Affiliations:** 1 Cancer Research UK, Institute for Cancer Studies, School of Cancer Sciences, University of Birmingham, Vincent Drive, Edgbaston, Birmingham, B15 2TT, UK; 2 Department of Innovative Surgery, Graduate School of Medicine, Mie University, 2-174, Edobashi, Tsu, Mie, 514-8507, Japan; 3 Department of Surgery, Toyama Hospital, 17-22, Minami-Schinmachi, Tsu, Mie, 514-0043, Japan; 4 Department of Gastrointestinal and Pediatric Surgery, Graduate School of Medicine, Mie University, 2-174, Edobashi, Tsu, Mie, 514-8507, Japan

**Keywords:** gastric, biomarker, serum, proteome

## Abstract

**Background:**

Proteomic methods have the potential to meet the urgent need for better cancer biomarkers. We have used a range of proteomic analyses of serum and tissue from gastric cancer patients and relevant controls to discover biomarkers for gastric cancer.

**Methods:**

Surface-enhanced laser desorption/ionisation time-of-flight mass spectrometry (SELDI) and antibody arrays were used to compare protein expression in 21 pairs of gastric cancer tissue and adjacent normal mucosa and serum from 51 gastric cancer patients and 29 patients with benign gastric diseases. Expression differences were confirmed by enzyme-linked immunosorbent assay.

**Results:**

Tissue analysis shows human neutrophil peptides 1–3 (HNPs 1–3) elevated 10-fold (*P*=0.001) in gastric cancer relative to adjacent normal mucosa. Macrophage migration inhibitory factor (MIF) was increased five-fold (*P*=1.84 × 10^−7^) in the serum of gastric cancer patients relative to individuals with benign gastric disease. The large increase in MIF concentration in serum gives an area under the receiver operating characteristic curve of 0.85.

**Conclusions:**

Proteomic analyses of serum and tissue indicate that HNPs 1–3 and MIF have potential as biomarkers for gastric cancer. In particular MIF may be useful, either alone or in combination with other markers, for diagnosing and monitoring gastric cancer.

Gastric cancer is the fourth most common cancer and the second leading cause of cancer-related death worldwide, surpassed only by lung cancer (Alberts *et al*, 2003; Kobayashi *et al*, 2004). Typically, gastric cancer is diagnosed at an advanced stage. Outcome depends on tumour stage at the time of diagnosis, with localised disease showing 62% 5-year survival, decreasing to 22% after spread to regional lymph nodes and to 3% with distant organ metastases (Jemal *et al*, 2007). Endoscopic examination is the most reliable method for the diagnosis of gastric cancer. Endoscopic screening is practiced widely in Japan because of the high incidence rate of gastric cancer but the feasibility and cost-effectiveness of this invasive approach in most countries remain questionable because of the lower incidence rates (Genta, 2004). A simple diagnostic test, such as a serum biomarker assay might facilitate screening for gastric cancer.

Currently, there are no serum biomarkers that are sufficiently sensitive and specific for routine diagnosis of gastric cancer (Marrelli *et al*, 1999, 2001; Gaspar *et al*, 2001; Ishigami *et al*, 2001). Although the combination of *Helicobacter pylori* serology and serum pepsinogen levels helps to identify a subgroup of individuals at higher risk for developing cancer (Varis *et al*, 2000; Bodger *et al*, 2001), it fails to differentiate tumour patients from non-tumour controls (Miki *et al*, 2003). The sensitivities of tumour markers such as CEA, CA 19–9 and CA 72–4 are low (20–30%) (Japanese Gastric Cancer Association, 1998; Gaspar *et al*, 2001; Ishigami *et al*, 2001; Marrelli *et al*, 2001).

Proteomic approaches can characterise hundreds–thousands of proteins in clinical samples and reveal novel alterations in protein structure or abundance with biomarker potential. It is hypothesised that tumours leak, shed and secrete proteins into the bloodstream, but the effects of dilution, expression in non-tumour tissues and the masking effect of abundant serum proteins make detection difficult. One approach that may overcome such limitations is to examine both serum and tissue by detecting and identifying differentially expressed proteins in tissue (using mass spectrometry-based approaches) and then quantifying these in serum with antibody-based tests.

In the experiments reported here, we use both surface-enhanced laser desorption/ionisation time-of flight mass spectrometry (SELDI) and antibody array analyses of tumour tissue homogenates and serum to identify novel biomarkers for gastric cancer. SELDI combines on-chip retentate chromatography with mass spectrometry to generate ‘proteomic profiles’ and can be applied to fluids, such as serum, plasma and urine and tissue extracts (e.g., Rogers *et al*, 2003; Albrethsen *et al*, 2005; Ward *et al*, 2006, 2008; Brozkova *et al*, 2008). SELDI provides a wealth of information on the low molecular proteome (<20 kDa), which contains diagnostic information (Villanueva *et al*, 2006). We now use this technique to analyse paired tissue and serum from gastric cancer patients and find that SELDI detects clear differences in protein levels between gastric tumours and normal mucosa. Three of the elevated SELDI peaks were identified as human neutrophil peptides 1–3 (HNPs 1—3). Antibody arrays detect macrophage migration inhibitory factor (MIF) as elevated in the serum of gastric cancer patients. We have assayed HNPs and MIF by enzyme-linked immunosorbent assay (ELISA) in tissue and serum to assess their potential as gastric cancer biomarkers.

## Materials and methods

### Sample collection

Samples were collected prospectively from October 2007 to March 2008, specifically for this project, from patients attending the Mie University Hospital and the Toyama Hospital in Japan. All patients gave informed consent for donating blood and tissue, and the study procedure was approved by the Institutional Review Board of the Mie University and the Toyama Hospital of Japan.

Gastric cancer tissue samples and matched adjacent normal gastric mucosa (*n*=21) were obtained after surgical resection, snap frozen in liquid nitrogen and stored at −80°C. The tumour/normal status of the tissue samples used for protein extraction was verified histologically. Most tumours were classified as pT2 and pT3. The 21 gastric cancer tissue samples were classified into various pathological stages according to the criteria of the Japanese Research Society for Gastric Cancer (Japanese Gastric Cancer Association, 1998) (Table 1).

Sera were obtained from 51 gastric cancer patients (Table 1). The control group comprised of 29 patients with gastritis or gastric ulcers diagnosed at endoscopy. All blood samples were collected in a fasting state in the early morning before surgery or medical treatment. Blood was allowed to clot at room temperature for 1–2 h, centrifuged at 3000 **g** for 20 min and the serum collected and stored at −80°C until processing.

### Preparation of tissue samples

Tissue samples were homogenised on ice in 100 *μ*l of 8 M urea +2% CHAPS containing protease inhibitor cocktail (Roche Diagnostics, Mannheim, Germany). The tissue homogenates were centrifuged at 12 000 **g** for 15 min to remove particulate material and stored at −80°C until use. The protein concentrations of the supernatants were determined using the Pierce BCA Protein Assay Kit (Thermo Fisher Scientific, Loughborough, UK) calibrated with bovine serum albumin.

### SELDI

All samples were analysed in duplicate on CM10 and Cu^2+^-loaded IMAC ProteinChip arrays using a PBS IIc time-of-flight mass spectrometer equipped with an autoloader (Ciphergen Biosystems Inc., Fremont, CA, USA). Serum samples were initially diluted 15-fold and tissue extracts to 1.5 mg protein per ml in 8 M urea +1% CHAPS. These diluted samples were further diluted 1 : 10 (serum) or 1 : 5 (tissue) in binding buffer (CM10: 0.1 M sodium acetate, pH 4.0, IMAC: 0.5 M NaCl, 0.1 M sodium phosphate, pH 7.0) and 100 *μ*l applied to the ProteinChip arrays. After 30 min incubation the chips were washed with binding buffer, rinsed with deionised water, air-dried and 1 *μ*l of 50% saturated sinapinic acid in 50% acetonitrile, 0.5% trifluoroacetic acid applied twice. Spectra were collected over m/z 0–20 000 and 0–200 000 ranges. Spectra were normalised to total ion current and baselines subtracted using Ciphergen ProteinChip software version 3.1 (Ciphergen Biosystems, Inc, Fremont, CA, USA). Peaks were detected and clustered using Biomarker Wizard software (default settings except for a requirement for a peak to be detected in more than 5% of the samples) (Ciphergen Biosystems Inc).

### Antibody arrays

The Panorama Antibody Microarray-XPRESS Profiler 725 kit (Sigma-Aldrich, Saint Louis, MO, USA) was used to compare pooled gastric cancer tissue extract (containing tissue extracts from 17 individuals, stages II–IV) and pooled normal gastric mucosa extract from the same 17 individuals. The experiment was conducted according to the manufacturer's instructions. Image acquisition and analysis was performed using an Axon GenePix 4000B laser scanner and GenePix 5.0 software package (Axon Instruments, Foster City, CA, USA). Background subtracted data were normalised to the median of a set of housekeeping genes using BRB-ArrayTools from the NCI, Bethesda, MD, USA (http://linus.nci.nih.gov/BRB-ArrayTools.html). Log2 ratios of the same antibody on two dye-swapped microarrays were averaged to eliminate dye effects.

The Proteome Profiler Human Cytokine Array Panel A (R&D Systems, Minneapolis, MN, USA) was used to compare pooled sera from 10 non-cancer controls, 10 patients with stage I and II gastric cancer and 10 patients with stage III and IV gastric cancer. Analysis of 200 *μ*l of each pool was carried out according to the manufacturer's instructions. Images were captured on X-ray films and digitised using a BioRad GC710 scanner. Spot intensities were then compared using PDQuest software (BioRad, Hemel Hempstead, UK).

### Identification of SELDI peaks with biomarker potential

Polypeptides were purified by anion exchange chromatography and reverse-phase high-performance liquid chromatography (HPLC) monitored by SELDI as described earlier (Ward *et al*, 2006). The HPLC fractions containing the polypeptides of interest were lyophilised, reduced with dithiothreitol (DTT), alkylated with iodoacetamide and subjected to LC-MS/MS analysis using a 75 *μ*m × 10 cm C8 column (New objective Woburn, MA, USA) and a 40 min gradient from 0 to 40% acetonitrile for peptide separation and a ThermoFinnigan LTQ-FT (Thermo Scientific Waltham, MA, USA) mass spectrometer. The CID MS/MS data were searched against the NCBI human database using Sequest within the Bioworks Browser software package (version 3.3.1; Thermo Scientific Waltham, MA, USA) using ‘no enzyme’. For all identifications, the precursor ion mass was within 10 p.p.m. of the predicted mass and the probability of an incorrect database match was <0.05.

### Quantitation of *H. pylori* IgG, CEA, HNPs 1–3 and MIF

The concentrations of *H. pylori* IgG, CEA, HNPs 1–3 and MIF were measured in duplicate by sandwich ELISA (*H. pylori* IgG; Demeditec Diagnostics GmbH, Keil-Wellsee, Germany, CEA; Fujirebio Diagnostics, Göteborg, Sweden, HNPs 1–3; Hycult Biotechnology, Uden, The Netherlands, MIF; R&D Systems) according to the manufacturer's instruction.

### HNPs 1–3 immunohistochemistry

Paraffin-embedded sections (5 *μ*m) of gastric cancer tissue and adjacent normal gastric epithelium were deparaffinised and dehydrated. The sections were microwaved in 10 mmol l^−1^ citric acid, pH 6.0, to inhibit endogenous peroxidase activity, rinsed twice with tris buffered saline (TBS), pH 7.6, and incubated overnight at 4°C in a humidity chamber with monoclonal antibody against HNPs 1–3 (T-1034; BMA Biomedicals; Augst, Switzerland 1 : 200 dilution). The tissue slices were then incubated with biotinylated goat antimouse immunoglobin followed by streptavidin-HRP. Finally, the sections were developed with diaminobenzidene-hydrogen peroxidase substrate (DAB; Dako Corporation Carpinteria, CA, USA), and lightly counterstained with haematoxylin.

### Statistical analysis

Wilcoxon tests, multiple regression analysis and generation of receiver operating characteristic (ROC) curves were performed using R (http://www.rproject.org). Paired Wilcoxon tests were used to analyse the tissue data and unpaired Wilcoxon tests to analyse the serum data. In addition, multiple regression analysis linear modelling (LM) was used to analyse the serum data to identify proteomic features significantly associated with cancer rather than age, gender or *H. pylori* level. For each proteomic feature, a linear model was fitted where intensity was explained by tumour status, age, gender and *H. pylori* IgG level. For each putative marker, ROC curves were generated to evaluate their discriminatory power. Partial least squares (PLS) regression was performed using PLS_Toolbox (Version 3.5, Eigenvector Research, Manson, WA, USA) running in Matlab (Version 7.1, The MathWorks, Natick, MA, USA).

## Results

### Tissue proteomics

**Figure 1:**
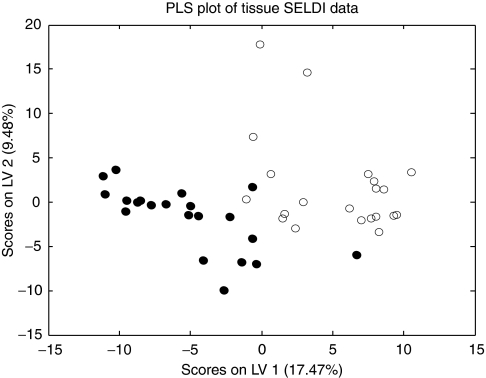
Partial least squares regression analysis using all SELDI peaks from 21 pairs of tumour/non-tumour tissue (CM10 and IMAC data). Tumour tissue is indicated by filled symbols and normal mucosa by hollow symbols.

The SELDI spectra of the tissue extracts contained 255 peaks (132 on IMAC30 and 123 on CM10). Comparison of SELDI spectra of 21 gastric cancer tissue extracts with paired adjacent normal mucosa showed a total of 115 differentially expressed protein peaks (*P*<0.05). A total of 65 peaks were upregulated in gastric cancer, whereas 50 were downregulated. The 10 most significant peaks are shown in Table 2. This data can be used to distinguish between gastric cancer and normal gastric mucosa as demonstrated by the PLS analysis shown in Figure 1.

A distinctive triplet of peaks were among the most significantly elevated peaks in the SELDI spectra of tumour tissue using both CM10 (m/z 3374, 3446 and 3490) and IMAC (m/z 3375, 3447 and 3492) ProteinChip arrays. A triplet of peaks with m/z values of 3372, 3443 and 3486, which bind to IMAC ProteinChip arrays, has previously been identified as HNPs 1–3 (Albrethsen *et al*, 2005). It seemed highly likely that our differentially expressed peaks also corresponded to HNPs 1–3 and this was confirmed by purification and LC-MS/MS of the intact polypeptides (Supplementary information, Figure S1 and Table S1).

**Figure 2:**
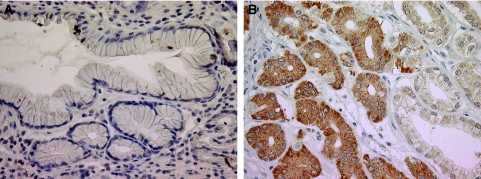
Human neutrophil peptides 1–3 expression in gastric epithelial cells. Immunostaining demonstrated negative expression in normal mucosa (**A**) and strong cytoplasmic expression in cancerous mucosa (**B**). Original magnification: × 400.

We used immunohistochemistry to confirm the overexpression of HNPs 1–3 in gastric tumours and to determine the site of expression. Human neutrophil peptides 1–3 expression was examined in gastric cancer tissues and adjacent normal tissue from five patients. In all cases, the normal gastric epithelium was negative for HNPs 1–3, whereas the tumour cells showed strong immunoreactivity, as shown in Figure 2.

Pooled gastric cancer tissue extract from 17 individuals and pooled normal gastric mucosa extract (from the same individuals) were analysed using an antibody microarray containing 725 different antibodies representing families of proteins known to be involved in a variety of different biological pathways. We found small changes in the levels of 24 proteins: 17 upregulated (1.5–2-fold) and 7 downregulated (<0.7) in cancer (Table 3).

### Serum proteomics

**Figure 3:**
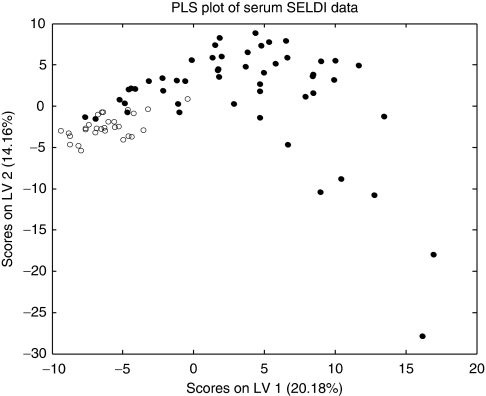
Partial least squares regression analysis using all peaks from CM10 and IMAC SELDI serum profiles of 50 gastric cancer patients (filled symbols) and 29 non-cancer controls (hollow symbols).

The SELDI spectra of serum contained 228 peaks (130 on IMAC, 98 on CM10). Partial least squares using all of the peaks can distinguish the serum of cancer and non-cancer patients (Figure 3). In addition to Wilcoxon tests, we used LM to investigate possible confounding factors. A total of 39 proteomic features were significantly associated with cancer in both statistical tests (*P*<0.05), but not significantly associated with age, gender or *H. pylori* IgG in LM (*P*>0.05). The 10 most significant peaks are shown in Table 4. We have used LC-MS/MS to identify three of the peaks that are substantially elevated in gastric cancer patients as fragments of inter-alpha-trypsin inhibitor heavy chain 4 (ITIH4). The MS/MS identifications are shown in ‘Supplementary information, Figure S2 and Table S1’. The IMAC peak at m/z 3030 corresponds to the peptide FRPGVLSSRQLGLPGPPDVPDHAAYHPF, the IMAC peak at m/z 3291 corresponds to M^*^NFRPGVLSSRQLGLPGPPDVPDHAAYHPF and the peak detected at m/z 4299 on IMAC chips and m/z 4303 on CM10 chips corresponds to NVHSAGAAGSRM^*^NFRPGVLSSRQLGLPGPPDVPDHAAYHPF (^*^denotes methionine oxidation).

**Figure 4:**
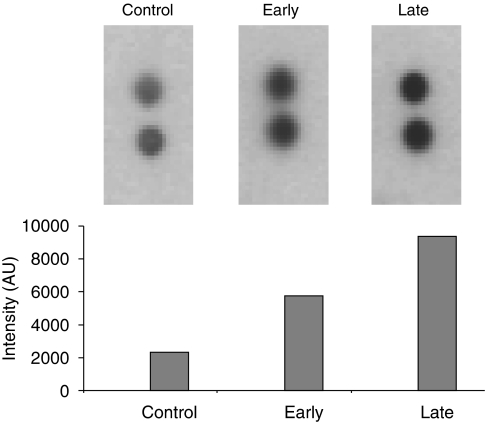
The images of the duplicate MIF antibody spots on the cytokine array for pooled control serum, pooled early (stages I–II) and pooled late (stages III–IV) sera. The quantification of the spot intensities is shown in the histograms.

Pooled serum from 10 individuals without cancer, early-stage gastric cancer (10 individuals, stages I–II) and late-stage gastric cancer (10 individuals, stages III–IV) were analysed using an array of antibodies to 36 cytokines. Most of the cytokines measured did not vary substantially between the three pooled samples, but the levels of MIF in this semi-quantitative assay were ∼2-fold increased in early-stage gastric cancer and ∼4-fold in late-stage gastric cancer compared with the control pool (Figure 4).

### ELISA of candidate biomarkers

**Figure 5:**
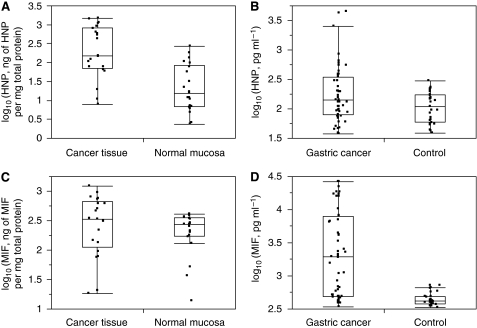
Panels **A** and **B** show the tissue and serum levels of HNPs 1–3, respectively, and panels **C** and **D** the tissue and serum levels of MIF, respectively. Data is plotted in log_10_ scale. The bar near the middle of the box represents the median value. The bottom and top of the box represent the lower and upper quartiles, respectively.

To confirm the SELDI data, HNPs 1–3 levels were determined by ELISA. The concentration of HNPs 1–3 in gastric cancer tissue was significantly higher than in adjacent normal gastric mucosa (median fold change=9.79; *P*=0.001, paired Wilcoxon's test)(Figure 5A). In the serum samples, the median concentration of HNPs 1–3 in gastric cancer patients was also higher than in controls (139 *vs* 108 pg ml^−1^), although this trend did not reach statistical significance (*P*=0.057, Wilcoxon's test) (Figure 5B). LM analysis produced *P*-values much greater than 0.05 for any possible associations between the serum level of HNPs 1–3 and age, gender or *H. pylori*.

The median concentration of MIF in gastric cancer patients’ serum was 1933 pg ml^−1^ compared with 414 pg ml^−1^ for the non-cancer controls (*P*=1.84 × 10^−7^, Wilcoxon's test) (Figure 5D). The tissue concentration of MIF, as determined by ELISA, was also elevated in gastric cancer tissue than in adjacent normal gastric mucosa (median fold change=1.33), although not statistically significant (*P*=0.082, paired Wilcoxon's test) (Figure 5C). As with HNPs 1–3, LM analysis indicated that serum MIF is not significantly influenced by gender, age or *H. pylori*.

### Assessment of biomarker potential

**Figure 6:**
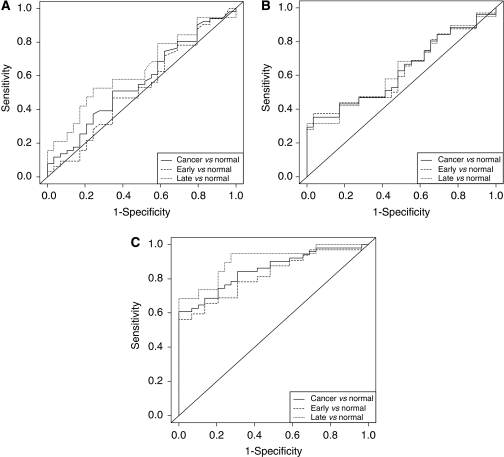
Receiver operator characteristic curves for CEA (**A**), HNPs 1–3 (**B**) and MIF (**C**). Curves for cancer *vs* control are shown as solid lines, early cancer *vs* control as dashed lines and late cancer *vs* control as dotted lines. The areas under the ROC curves are for CEA: 0.567, 0.526 and 0.636, HNPs 1–3: 0.629, 0.628 and 0.630, MIF: 0.853, 0.820 and 0.907 (non-cancer *vs* all cancer, early cancer and late cancer, respectively).

Receiver operating characteristic curves for the discrimination between gastric cancer patients and the non-cancer controls were constructed based on serum levels of CEA, HNPs 1–3 and MIF (Figure 6). The areas under the ROC curves were 0.57, 0.63 and 0.85. At a threshold of 800 pg ml^−1^, MIF correctly detects 31 out of 51 cancers (61% sensitivity) and all non-cancers (100% specificity). Combining CEA and HNPs 1–3 with MIF did not substantially increase the area under the ROC curve (data not shown).

## Discussion

Proteomic analyses of serum and tissue samples from patients with gastric cancer and appropriate controls have shown HNPs 1–3 and MIF as elevated in gastric cancer. Human neutrophil peptides 1–3 are substantially elevated in gastric cancer tissue (as shown by SELDI tissue analysis and confirmed by ELISA and ELISA) and MIF is substantially elevated in the serum of gastric cancer patients (as shown by antibody array analysis of serum and confirmed by ELISA). We also found a number of SELDI peaks that differed significantly between the serum of cancer patients and controls and four of the peaks substantially elevated in gastric cancer have been identified as fragments of ITIH4. Interestingly, these and other fragments of ITIH4 have previously been found to be up or downregulated in the serum of patients with various cancers and this is believed to arise from disease associated alterations in protease activity (Koomen *et al*, 2005; Song *et al*, 2006; Villanueva *et al*, 2006). A commercially available high-density antibody microarray was used to analyse tissue protein levels (Table 3), but did not detect any proteins where the expression differences exceeded two-fold.

Human neutrophil peptides 1–3 are members of the α-defensin family of antimicrobial peptides reported to be expressed by neutrophils and epithelial cells under certain conditions (Cunliffe *et al*, 2002). Human neutrophil peptides 1–3 have been shown to be elevated in either tumour tissue or serum from patients with a range of cancer types (Müller *et al*, 2002; Lundy *et al*, 2004; Albrethsen *et al*, 2005; Melle *et al*, 2005), and we now extend this observation to gastric cancer. Human neutrophil peptides 1–3 are also elevated by inflammation, for example, inflammatory bowel disease (Cunliffe *et al*, 2002) and so may lack specificity as tumour markers. Melle *et al* (Melle *et al*, 2005) have presented data suggesting that the HNPs 1–3 are overexpressed by tumour cells in colon cancer, but Lundy *et al* (2004) reported abundant HNPs 1–3 in infiltrating neutrophils in oral cancer consistent with HNPs 1–3 playing a role in innate host defence against the tumour. We have used immunohistochemistry to localise the expression of HNPs 1–3 in gastric tissues. The data shown in Figure 2 clearly indicate that the source of elevated HNPs 1–3 in gastric cancers is expression by the epithelial cells of the tumours rather than by infiltrating neutrophils.

Overexpression of MIF has been reported in prostate, breast, colon and hepatocellular carcinomas (Akbar *et al*, 2001; Meyer-Siegler *et al*, 2002; Lee *et al*, 2008; Xu *et al*, 2008). In 2006 He *et al*. (He *et al*, 2006) reported MIF overexpression in the tissue and serum of gastric cancer patients from Hong Kong. More recently Camlica *et al*. (Camlica *et al*, 2008) reported elevated serum MIF in gastric cancer patients relative to healthy controls. We now report that this is also the case in patients from Japan, a country with one of the highest incidences of gastric cancer in the world (Parkin *et al*, 2005), and add to the body of evidence that MIF may be a useful biomarker for gastric cancer. Furthermore, our data suggest that serum MIF is elevated in the early stages of gastric cancer relative to relevant disease control subjects, a factor that is of considerable importance in establishing a useful diagnostic role for this potential biomarker. In agreement with the findings of He *et al* (2006), we find that tissue and serum levels of MIF are not strongly influenced by *H. pylori.*

Macrophage migration inhibitory factor is a pro-inflammatory cytokine, which is able to promote tumour cell proliferation, migration and metastasis and tumour angiogenesis (Wilson *et al*, 2005; Xu *et al*, 2008). Mechanisms involved include activation of the MAP kinase pathways through CD74 and CD44 (Shi *et al*, 2006), suppression of p53 (Hudson *et al*, 1999; Fingerle-Rowson *et al*, 2003) and downregulation of NKG2D enhancing immune evasion by cancer cells (Krockenberger *et al*, 2008). As with HNPs 1–3, MIF may lack specificity for gastric cancer as it has been reported as elevated in the plasma of patients with ulcerative colitis and Crohn's disease (de Jong *et al*, 2001; Murakami *et al*, 2001). However, preliminary work in our laboratory suggests a degree of disease specificity: serum MIF is also elevated in European patients with hepatocellular carcinoma (relative to our 29 Japanese non-cancer controls), but is not elevated in patients with lung or pancreas cancer or individuals without cancer (n ⩾ 30 per group, data not shown).

In conclusion, our proteomic analyses of tissue and serum from gastric cancer patients have shown MIF, HNPs 1–3 and fragments of ITIH4 as potential biomarkers for gastric cancer. In particular, serum MIF is highly elevated in the potentially curable early stages of gastric cancer thus warranting further studies to validate this candidate biomarker as a blood test for gastric cancer, either on its own or as part of a panel of biomarkers.

## Figures and Tables

**Table 1:** Demographic and clinical features of patients from whom tissue and serum samples were collected

**Tissue samples**		**Serum samples**	
Age (mean, range)	71.1, 58–81 years	Gastric cancer (*n*=51)	
Male/female	16/5	Age (mean, range)	65.9, 37–87 years
Stage I	4	Male/female	41/10
Stage II	1	Stage I	25
Stage III	4	Stage II	7
Stage IV	12	Stage III	4
		Stage IV	15
		*H. pylori* (mean, range)	41.3, 5.0–138.4
		Gastric ulcer/gastritis (*n*=29)	
		Age (mean, range)	40.0, 22–69 years
		Male/female (*n*)	17/12
		*H. pylori* (mean, range) (U ml^−1^)	23.4, 4.7–133.4

**Table 2:** The 10 most significantly cancer associated SELDI peaks in the tissue analysis

**Peak**	***P*-value (Wilcoxon's test)**	**Intensity ratio (C/N)**
CM10 mz 2491	4.77 × 10^−6^	0.166
IMAC mz 7475	6.68 × 10^−6^	0.278
IMAC mz 3375^*^	9.54 × 10^−6^	2.164
CM10 mz 4039	1.81 × 10^−5^	0.176
IMAC mz 3447^*^	2.38 × 10^−5^	1.998
CM10 mz 5836	2.38 × 10^−5^	3.006
CM10 mz 3446^*^	3.15 × 10^−5^	2.962
CM10 mz 2380	3.15 × 10^−5^	0.184
IMAC mz 2515	4.10 × 10^−5^	0.254
CM10 mz 6563	4.10 × 10^−5^	2.596

For each peak, we show the *P*-value (Wilcoxon's test) and the ratio of the median peak height in cancer patients divided by the median peak height in non-cancer controls. The peaks marked with asterisks are HNP 1 (molecular weight=3448) and HNP 2 (molecular weight=3377).

**Table 3:** Proteins showing greater than 1.5-fold or less than 0.7-fold altered expression in tumour tissue relative to adjacent normal tissue on the Panorama array

**Antibody**	**Gene symbols**	**Function**	**Ratio (C/N)**
Proliferating cell nuclear antigen	*PCNA*	Gene regulation and expression	1.91
Heat-shock factor 1	*HSF1*	Cell stress	1.86
BACH1	*BRIP1*	Gene regulation and expression	1.8
BAD	*BAD*	Apoptosis	1.79
Nitric oxide synthase, brain (b-NOS)	*NOS1*	Neurodegenerative diseases	1.71
MAP1 (Light chain)	*Map1lc3a*	Neuronal development	1.7
Tropomyosin	*TPM1*	Cytoskeleton	1.69
NG2	*Cspg4*	Neuronal development	1.65
Transforming growth factor-?, pan	*TGFB3, TGFB1, TGFB2*	Kinase/phosphatase biology	1.62
Cytokeratin 8.12	*KRT15, KRT16, KRT13*	Cytoskeleton	1.6
Calmodulin	*Calm1*	Intracellular calcium signalling	1.59
Rad17 (C-terminal)	*RAD17*	Cell cycle	1.57
Importin-a3	*KPNA4*	Gene regulation and expression	1.56
MBD1	*MBD1*	Gene regulation and expression	1.56
MRP2	*ABCC2*	Multidrug resistance	1.54
MBNL 1	*MBNL1*	Gene regulation and expression	1.53
Rab9	*RAB9*	G proteins and cyclic nucleotides	1.53
F1A	*FEM1B*	Apoptosis	0.66
PUMA/bbc3, C-terminal	*BBC3*	Cell cycle	0.66
DR5	*TNFRSF10B*	Apoptosis	0.65
PID/MTA2	*MTA2*	Gene regulation and expression	0.64
p53DINP1/SIP	*TP53INP1*	Cell cycle	0.64
MAP kinase 2 (ERK-2)	*MAPK1*	Kinase/phosphatase biology	0.6
p53R2l	*RRM2B*	Cell cycle	0.6

‘Function’ was provided by Sigma-Aldrich and gives an indication of the role of each protein. The experiment was conducted using pooled tissue extracts as described in the Materials and Methods section.

**Table 4:** The 10 most significant cancer-associated SELDI peaks in the serum analysis

**Peak**	***P*-value (Wilcoxon's test)**	**Ratio (C/N)**	**ROC area**
CM10 mz 6679	3.50 × 10^−8^	0.704	0.873
CM10 mz 4303#	3.92 × 10^−8^	5.759	0.872
IMAC mz 9632	2.41 × 10^−7^	0.682	0.849
IMAC mz 3030#	3.69 × 10^−7^	6.139	0.844
IMAC mz 133000	1.04 × 10^−6^	0.614	0.830
IMAC mz 3291#	1.34 × 10^−6^	8.355	0.827
IMAC mz 4299#	2.31 × 10^−6^	7.655	0.819
IMAC mz 6629	2.43 × 10^−6^	0.685	0.819
IMAC mz 3956	2.43 × 10^−6^	4.266	0.819
IMAC mz 9705	2.68 × 10^−6^	0.698	0.817

We present *P*-value for cancer *vs* non-cancer (Wilcoxon's test), cancer median peak height divided by non-cancer median peak height and area under the ROC curve. All of these peaks were also significant by LM and were not significantly associated with age, gender or *H. pylori* (*P*>0.05). Peaks arising from fragments of ITIH4 are marked with #.
