# Supplementary material for: Identification of macrophage migration inhibitory factor and human neutrophil peptides 1–3 as potential biomarkers for gastric cancer
Source: Br J Cancer. 2009 Jun 23;101(2):295–302. doi: 10.1038/sj.bjc.6605138 (PMC2720195; doi:10.1038/sj.bjc.6605138)
Supplement: Supplementary Figures S1 and S2 [file 6605138x1.doc]

Figure S1 shows the MS/MS spectra used to identify HNPs 1, 2 and 3.

Figure S2 shows MS/MS spectra used to identify three fragments of ITIH4.
