# Supplementary material for: Identification of macrophage migration inhibitory factor and human neutrophil peptides 1–3 as potential biomarkers for gastric cancer
Source: Br J Cancer. 2009 Jun 23;101(2):295–302. doi: 10.1038/sj.bjc.6605138 (PMC2720195; doi:10.1038/sj.bjc.6605138)
Supplement: Supplementary Table S1 [file 6605138x2.doc]

| Name | Sequence | M(H^+^) | Charge | Δppm | Xcorr | P | +1 (b,y) | +2 (b,y) | +3 (b,y) | +4 (b,y) | Comments |
| --- | --- | --- | --- | --- | --- | --- | --- | --- | --- | --- | --- |
| HNP-1 | ACYCRIPACIAGERRYGTCIYQGRLWAFCC | 3788.69 | 4 | -1.7 | 2.7 | 8 x 10^-7^ | 10, 12 | 7, 5 | 8, 5 | 4, 7 | Found x3 |
| HNP-2 | CYCRIPACIAGERRYGTCIYQGRLWAFCC | 3717.66 | 4 | -4.7 | 2.7 | 6 x 10^-5^ | 6, 8 | 9, 10 | 9, 6 | 6, 4 | Found x3, 5^+^ once |
| HNP-3 | DCYCRIPACIAGERRYGTCIYQGRLWAFCC | 3832.68 | 4 | -7.0 | 1.8 | 5 x 10^-2^ | 8, 10 | 8, 7 | 8, 7 | 4, 6 | Found once |
| ITIH4 | FRPGVLSSRQLGLPGPPDVPDHAAYHPF | 3027.26 | 4 | -4.7 | 3.2 | 3 x 10^-9^ | 3, 12 | 13, 6 | 4, 4 | 2, 3 | Found x2, 5^+^ once |
| ITIH4 | M*NFRPGVLSSRQLGLPGPPDVPDHAAYHPF | 3288.64 | 5 | -4.3 | 2.7 | 5 x 10^-7^ | 4, 7 | 9, 7 | 6, 4 | 1, 3 | Also 3^+^ x 2, 4^+^ once |
| ITIH4 | NVHSAGAAGSRM*NFRPGVLSSRQLGLPGPPDVPDHAAYH | 4296.13 | 6 | -4.6 | 2.1 | 5 x 10^-5^ | 5, 7 | 7, 10 | 11, 5 | 7, 2 | Also 4^*^ once, 5^+^ once |

Table S1. LC-MS/MS polypeptide information. The table summarises the database search results used to identify HNP1-3 and the fragments of ITIH4 underlying SELDI peaks of interest. We show the sequence and theoretical mass of the monoisotopic H^+^ peptide ion, the charge on the peptide, Δppm indicates the discrepancy between the measured and theoretical peptide masses in parts per million, Xcorr is an indication of how well the fragmentation data matches theoretical fragmentation data for that peptide, P is the probability of an incorrect database match and we also show the number of singly, doubly, triply and quadruply charged b and y fragment ions and report how many times each peptide match was obtained.
